# Spinal epidural lipomatosis

**DOI:** 10.1186/s12245-021-00404-2

**Published:** 2022-03-24

**Authors:** Hanae Ramdani, Manal Jidal, Rachida Saouab, Imad-Eddine Sahri, Hassan En-Nouali, Jamal El Fenni

**Affiliations:** 1 Radiology Department, Mohammed Vth military hospital, Ryad street, 10010 Rabat, Morocco; 2 Neurosurgery Department, Mohammed Vth military hospital, Ryad street, 10010 Rabat, Morocco

## Abstract

Spinal epidural lipomatosis (SEL) is a rare pathologic growth of histologically normal nonencapsulated adipose tissue in the epidural space. It can cause myelopathy or radiculopathy. Etiologies include chronic exposure to endogenous or exogenous steroids and obesity. Idiopathic forms are much infrequent. We present a case of lumbar SEL compressing the thecal sac in a 50-year-old female patient.

A 50-year-old female patient presented to our radiology department complaining of lumbar and right lower extremity pain and weakness. Magnetic resonance imaging (MRI) of the lumbo-sacral spine demonstrated severe circumferential compression of the dural sac (from L5 to S1) caused by significant epidural fat hypertrophy (Fig. 1a–c). The compressed thecal sac adopted a “Y” configuration (Fig. 2), a finding characteristic of a grade III Borré et al. spinal epidural lipomatosis (SEL).

**Fig. 1:**
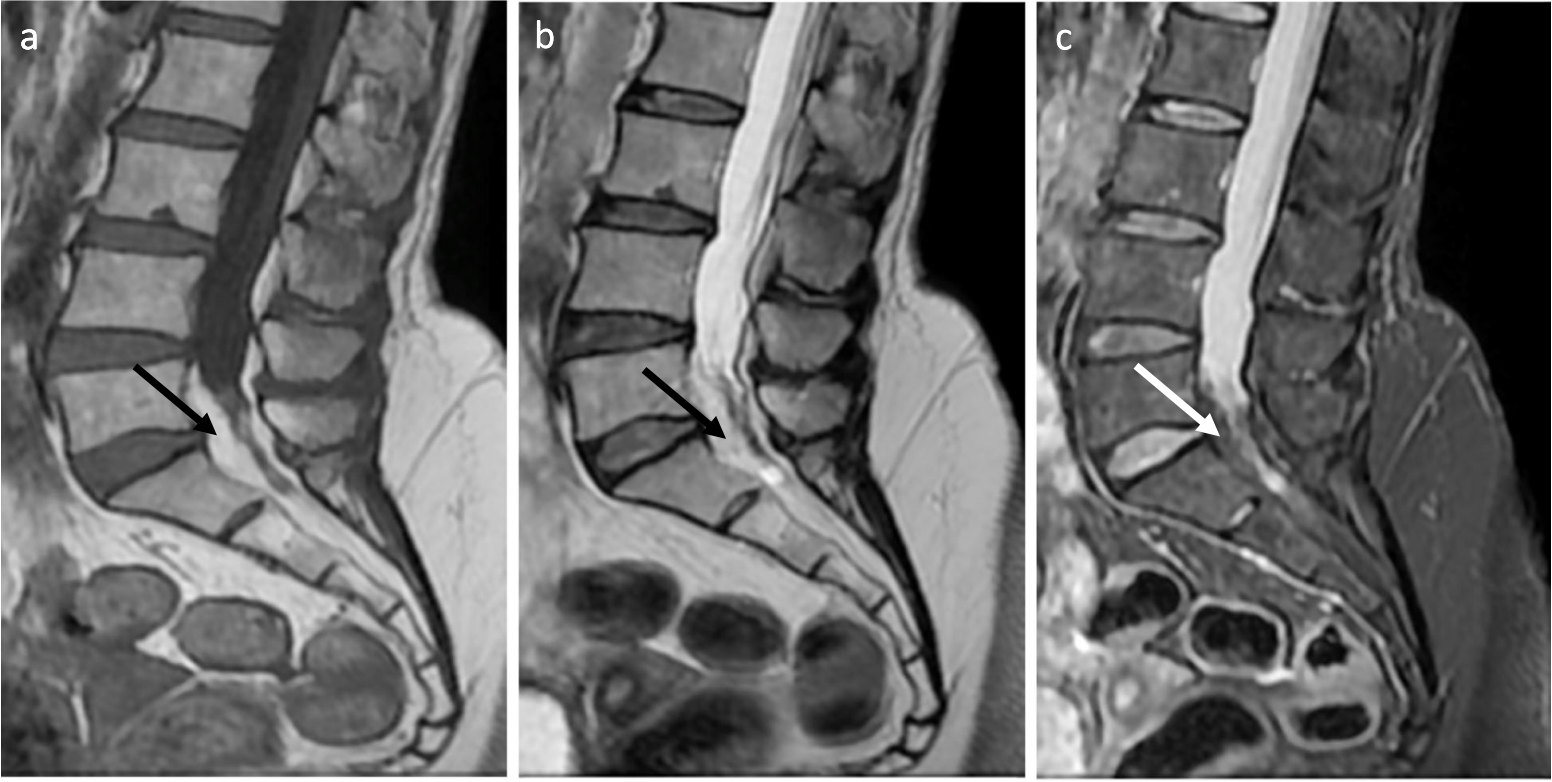
Midsagittal T1 (**a**), T2 (**b**), and STIR (**c**) weighted images show a large amount of circumferential epidural fat (black and white arrows) surrounding the dural sac at level L5-S1. No other substantial lumbar spine abnormality is present

**Fig. 2:**
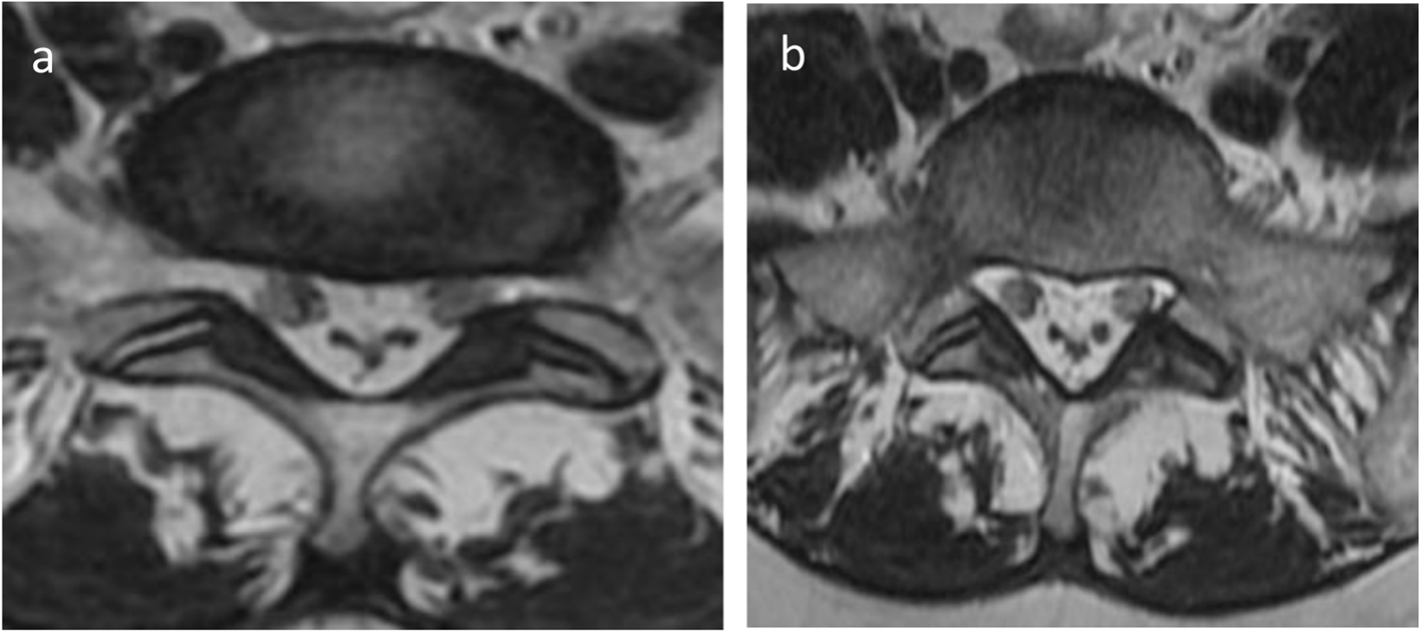
Axial T2-weighted images at L5-S1 interspace (**a**) and S1 superior end plate (**b**) demonstrate the pathognomonic “Y” shape of the dural sac

SEL is a rare condition characterized by nonencapsulated adipose tissue accumulation in the thoracic or lumbar spinal canal’s epidural space which can cause spinal cord or nerve root compression [1, 2]. It can be due to chronic steroid therapy (55% of cases), obesity (25%), Cushing’s syndrome (3%), or idiopathic (17%) [3]. According to recent studies, SEL should be contemplated as a metabolic syndrome manifestation, alongside increased BMI, abdominal circumference, and visceral and liver fat deposits [4]. To diagnose and grade this condition, MRI is the gold standard imaging modality. Mild (grade I) SEL is asymptomatic, moderate (grade II) SEL is symptomatic in 14.5% of cases, whereas all severe (grade III) SEL cases are symptomatic [1]. Weight loss and/or steroids suspension are efficient therapeutic strategies. When conservative approaches fail, surgical management involving decompressive laminectomy and epidural adipose tissue excision is indicated [5].

## Data Availability

Data are available on reasonable request from the authors.
